# LCASPMDA: a computational model for predicting potential microbe-drug associations based on learnable graph convolutional attention networks and self-paced iterative sampling ensemble

**DOI:** 10.3389/fmicb.2024.1366272

**Published:** 2024-05-23

**Authors:** Zinuo Yang, Lei Wang, Xiangrui Zhang, Bin Zeng, Zhen Zhang, Xin Liu

**Affiliations:** Big Data Innovation and Entrepreneurship Education Center of Hunan Province, Changsha University, Changsha, China

**Keywords:** prediction model, drug-microbe association, learnable graph convolutional attention network, self-paced iterative sampling ensemble, multi-layer perceptron classifier

## Abstract

**Introduction:**

Numerous studies show that microbes in the human body are very closely linked to the human host and can affect the human host by modulating the efficacy and toxicity of drugs. However, discovering potential microbe-drug associations through traditional wet labs is expensive and time-consuming, hence, it is important and necessary to develop effective computational models to detect possible microbe-drug associations.

**Methods:**

In this manuscript, we proposed a new prediction model named LCASPMDA by combining the learnable graph convolutional attention network and the self-paced iterative sampling ensemble strategy to infer latent microbe-drug associations. In LCASPMDA, we first constructed a heterogeneous network based on newly downloaded known microbe-drug associations. Then, we adopted the learnable graph convolutional attention network to learn the hidden features of nodes in the heterogeneous network. After that, we utilized the self-paced iterative sampling ensemble strategy to select the most informative negative samples to train the Multi-Layer Perceptron classifier and put the newly-extracted hidden features into the trained MLP classifier to infer possible microbe-drug associations.

**Results and discussion:**

Intensive experimental results on two different public databases including the MDAD and the aBiofilm showed that LCASPMDA could achieve better performance than state-of-the-art baseline methods in microbe-drug association prediction.

## 1 Introduction

The human body contains trillions of microbes, including bacteria, archaea, fungi, protozoa, and viruses, which constitute the human microbiota and interact closely with the human host (The Human Microbiome Project Consortium, 2012; Sommer and Bäckhed, 2013). These microbes can be found in the skin, oral cavity, nasal cavity, gastrointestinal tract, genitourinary tract and other parts of the human body, and play an important role in regulating human health. For example, they can regulate the pathology of the gastrointestinal tract and harmonize the homeostasis of the internal environment in order to promote the metabolic functions of the body (Gill et al., 2006; Ventura et al., 2009). The microbiome and host mucosal sites interact in a synergistic manner to protect against pathogens (Macpherson and Harris, 2004). Microorganisms promote the synthesis of sugar metabolism and facilitate the synthesis of vitamins required for t-cell reactions (Kau et al., 2011). But microorganisms also have adverse effects on the human body. For instance, studies have proved that dysbiosis of microbial communities can induce diabetes (Wen et al., 2008), inflammatory bowel disease (Durack and Lynch, 2019) and even cancer (Schwabe and Jobin, 2013). And additionally, pathogens such as bacteria and viruses have been proven to be able to cause as many as 27 infectious diseases such as COVID-19 (Xiang et al., 2020). Moreover, in recent years, due to the abuse and irrational use of drugs, microbes have developed resistance to some drugs, which has brought serious challenges to clinical medicine and drug development. In addition, recent studies have also shown that the efficacy of drugs is significantly influenced by the microbial metabolism (McCoubrey et al., 2022). When drugs are functioning in the human body, microorganisms play an important role in drug absorption and metabolism, thereby modulating drug efficacy and toxicity (Zimmermann et al., 2019). Concetta et al. reported that gut microbiota can interact with anticancer drugs, thus affecting the therapeutic efficiency and toxic side effects of drugs. They considered the probiotics, prebiotics, synbiotics, biologics and antibiotics as emerging strategies for microbiota control, which might improve treatment outcomes or ensure that patients have a better quality of life during anticancer treatment (Panebianco et al., 2018). Therefore, the discovery of potential microbial-drug associations is one of the key problems to be solved in the field of precision medicine, and the need to develop an efficient computational model to discover potential microbial-drug associations is becoming more and more urgent.

Since traditional wet tests are very expensive, time-consuming and inefficient, moreover, in recent years, the advances in bioinformatics technology have given birth to lots of public microbial drug association databases, including MDAD (Sun et al., 2018), aBiofilm (Rajput et al., 2018), and DrugVirus (Andersen et al., 2020), researchers have developed more and more feasible and efficient computational models based on these publicly available databases to infer potential microbe-drug associations (Long et al., 2022), which can be roughly divided into five main categories: network-based, matrix decomposition, matrix complementation, regularization and neural networks. For example, Zhu et al. (2019) designed a method called HMDAKATZ to detect latent associations between microbes and drugs by combining microbe-drug heterogeneous networks with the KATZ metrics. Long et al. proposed a prediction model named GCNMDA by adopting graph neural networks and conditional random fields with attentional mechanisms to learn deep representations of microbes and drugs (Long et al. 2020a), and a calculation model called EGATMDA (Long et al., 2020b) to predict potential associations between microorganisms and drugs by adopting a graph convolutional network with graph-level attention mechanism to learn the importance of different heterogeneous networks and a graph convolutional network with node-level attention to learn the embedding of nodes in the heterogeneous networks. Deng et al. (2022) devised a method called Graph2MDA to detect possible associations between microbes and drugs, in which, multimodal attribute maps were constructed as inputs of the variogram self-encoder to obtain informative and interpretable latent features of microbes and drugs. Tan et al. (2022) constructed a novel prediction model GSAMDA by integrating the graph attention network and the sparse self-encoder, in which, the graph attention network and the sparse self-encoder were adopted to extract topological features and node features of microbes and drugs in heterogeneous networks, respectively. Ma et al. (2023) employed a two-layer graphical attention network to learn the features of microbes and drugs, and subsequently adopted a convolutional neural network classifier to detect potential microbe-drug associations. MHBVDA combined two new methods, such as the Matrix Decomposition for Heterogeneous Graph Inference (MDHGI) and the Bounded Nucleus Paradigm Regularization (BNNR), to construct virus-drug heterogeneous networks by using multi-source heterogeneous data of viruses and drugs, and then reconstructed the adjacency matrix of the network to predict the missing virus-drug associations (Qu et al., 2023). NIRBMMDA first obtained two potential microbe-drug association matrices to calculate drug-microbe associations for similar drugs and microbe-drug associations for similar microbes by using different thresholds to find similar neighbors of drugs or microbes, respectively, and then obtained another potential microbe-drug association matrix based on the contrast scatter algorithm and the sigmoid function to learn the hidden probability distributions in the known microbe-drug associations (Cheng et al., 2022).

Although above methods can achieve excellent prediction performance, there still exist some limitations. For instance, HMDAKATZ uses only simple metrics to evaluate the strength of microbe-drug associations, EGATMDA only randomly selects negative samples while ignores the specificity of different negative samples. Besides, recent studies have shown that the performance of Graph Convolutional Networks (GCN) and Graph Attention Networks (GAT) depend on the nature of selected datasets (Knyazev et al., 2019; Baranwal et al., 2021; Fountoulakis et al., 2022), which means that the GCN-based GCNMDA cannot achieve satisfactory prediction on multiple different datasets at the same time, neither can the GAT-based GSAMDA. Therefore, in order to achieve better prediction performance, we need to choose between Graph Convolutional Networks (GCN) and Graph Attention Networks (GAT) through cross-validation. For this purpose, CAT (Graph Convolutional Attention Layer) is introduced to solve this problem. However, intensive experimental results have demonstrated that CAT can achieve better performance than both GAT and GCN at low noise levels in the dataset, but cannot improve the prediction performance significantly at higher noise levels, which means that there is no absolute difference between GCN, GAT and CAT, and their effectiveness is directly affected by the selected dataset. To solve this problem, Learnable Graph Convolutional Attention Networks (LCAT; Javaloy et al., 2023) came into existence. Through efficiently combining the different GNN layers by adding two scalar parameters that are automatically interpolated in each layer of GCN, GAT and CAT, LCAT outperforms the methods of GCN, GAT and CAT in a wide range of datasets. Hence, it is obvious that, if we employ LCAT in the prediction model to infer possible microbial-drug associations, we can not only achieve better performance but also subtract the cross-validation requirement of choosing between the methods of GCN, GAT and CAT.

Moreover, in binary relationship prediction, how to select negative samples is important for model training, but selecting informative negative samples from the set of candidate negative samples is still an intractable problem (Li et al., 2022). In link prediction problems, how to generate candidate negative samples has always been one of the challenges. Existing machine learning methods usually treat known associations between entities (labeled samples) as positive samples and unrecognized associations (unlabeled samples) as candidate negative samples (Yang et al., 2012). However, since the number of known microbe-drug associations is very small in existing public datasets, the proportion of positive and negative samples will be extremely unbalanced in this case. Therefore, in order to avoid extreme imbalance in the proportion of positive and negative samples affecting the performance of the prediction model, we need further to perform a negative under-sampling strategy for candidate negative samples. But, as for the negative under-sampling strategies, the most common method is the random sampling, i.e., a subset of negative samples with the same number as the set of positive samples will be randomly selected from the candidate negative samples (Lou et al., 2022). These random sampling-based strategies, while simple, tend to ignore informative negative samples and introduce less meaningful and noisy negative samples (López et al., 2013). Although there are some models that can improve the negative sampling strategy (Zeng et al., 2020; Wei et al., 2021; Dai et al., 2022), but they do not focus on filtering out the most informative negative samples that play an important role for the classifier during the model training process, which may lead to under-training of the model, thus limiting the predictive power of the model.

Based on above analysis, in this study, we proposed a novel computational model LCASPMDA by integrating the Learnable Graph Convolutional Attention network and the Self-Paced iterative sampling ensemble strategy to identify potential Microbe-Drug Associations. In LCASPMDA, we will first construct a heterogeneous network of microbes and drugs based on these newly downloaded known microbe-drug associations and an integrated similarity of microbes and drugs. And then, we will employ LCAT to learn the hidden feature representations of nodes from heterogeneous networks. Subsequently, we will introduce the self-paced iterative sampling ensemble scheme to train the MLP classifier by selecting the most informative negative samples based on the prediction results after each training of the model, and finally input the feature representations extracted by the LCAT into the trained MLP (Multi-Layer Perceptron) classifier to infer potential associations between microbes and drugs. Intensive experimental results on two well-known public datasets showed that LCASPMDA significantly outperformed state-of-the-art competitive prediction methods in the prediction task of latent microbe-drug associations. And in addition, case studies of two common drugs further demonstrated the superiority of LCASPMDA in discovering new microbe-drug associations as well.

## 2 Materials and methods

**Figure 1:**
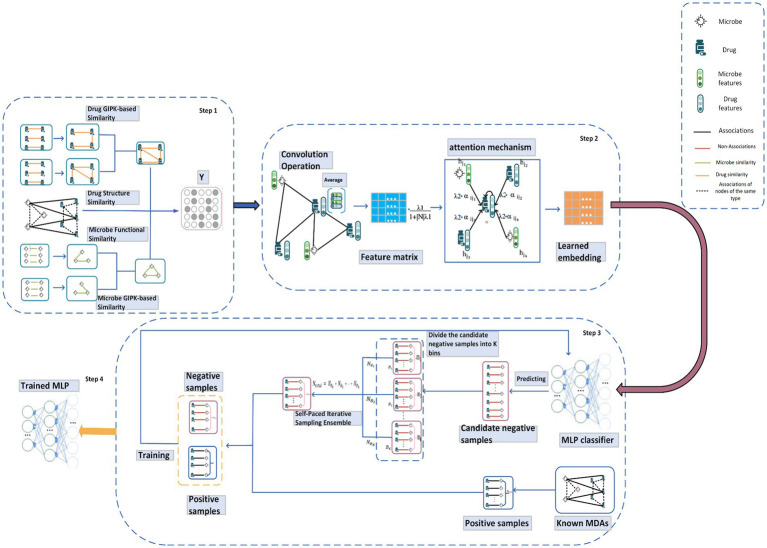
The overall framework of LCASPMDA. Step1: a heterogeneous network of microbes and drugs is constructed based on newly-downloaded known microbe-drug associations and an integrated similarity of microbes and drugs. Step2: the heterogeneous network is inputted into the LCAT to learn the feature representations of nodes. Step 3: The Self-Paced Iterative Sampling Ensemble is adopted to select the most informative samples for training the MLP classifier while ensuring the balance of training samples. Step 4: potential associations between microbes and drugs are inferred by the trained MLP.

As illustrated in Figure 1, LCASPMDA consists of four major steps. In the first step, we construct a heterogeneous network of microbes and drugs based on newly-downloaded known microbe-drug associations and an integrated similarity of microbes and drugs. In the second step, we adopt the LCAT to learn the feature representations of nodes in the heterogeneous network of microbes and drugs. In the third step, we introduce a Self-Paced Iterative Sampling Ensemble to select informative negative samples to train the MLP classifier. In the final step 4, we utilize the trained MLP to detect potential microbe-drug associations based on a novel loss function.

### 2.1 Datasets

In this section, we first downloaded known microbe-drug associations from the MDAD database, which were derived from 993 papers covering 1,388 drugs and 180 microorganisms (Wang et al., 2022). After de-duplication, we finally obtained 2,470 known microbe-drug associations involving 173 microbes and 1,373 drugs. Subsequently, we further downloaded known microbe-drug associations for validation from the aBioflm database, which contained 2,884 known microbe-drug associations between 1,720 drugs and 140 microbes. Additionally, we downloaded the dataset of DrugVirus from the research (Long et al., 2020a),^1^ in which, there are 95 microbes and 175 drugs including 933 microbe–drug associations between them. Table 1 illustrated the statistical information of these two kinds of newly downloaded datasets.

**Table 1:** The statistics of these two newly-downloaded databases.

Datasets	Microbes	Drugs	Associations
MDAD	173	1,373	2,470
aBiofilm	140	1,720	2,884
DrugVirus	95	175	933

In each dataset, we used an adjacency matrix to represent the association relationship between microorganisms and drugs. Without loss of generality, the adjacency matrix can be represented as $A\in R^{N_{d\;}\times N_{m}}$ , where $N_{m\;\;}\;$ and $N_{d}$ denote the number of microbes and drugs in the dataset, respectively. In the adjacency matrix *A*, for any given drug $d_{i}$ and microbe $m_{j}$ , if there is a known association between them, then the value of A(i,j) will be 1, otherwise the value of A(i,j) will be 0.

### 2.2 Construction of the heterogeneous network of microbes and drugs

#### 2.2.1 Calculation of the integrated similarity of microbe

In LCASPMDA, the similarity between microbes will be measured in two different ways. This first one is measured by the Gaussian interaction-profile-kernel similarity. Considering that drugs with similar therapeutic effects will be associated with similar microorganisms, let *C*(*i*) and *C*(𝑗) denote the *i*th and *j*th column of the adjacency matrix *A* separately, then for any two given microorganisms $m_{i}$ and $m_{j}$ , the Gaussian interaction-profile-kernel similarity between them can be computed as follows:


(1) $$Sm_{G}\left(m_{i},m_{j}\right)=\exp\left(-\mu_{m}{\left\|C\left(i\right)-C\left(j\right)\right\|}^{2}\right)$$

Where $\mu_{m}$ is the normalized kernel bandwidth, which is calculated as:


(2) $$\mu_{m}=\;\;\mu_{m}^{\prime }{\left(\frac{1}{N_{m}}{\sum_{i=1}^{N_{m}}\left\|C\left(i\right)\right\|}^{2}\right)}^{-1}$$

Here $\mu_{m}^{\prime }$ is the original bandwidth, which is usually set to 1. After determining the similarity of all microbial pairs according to above equations, then it is obvious that we can obtain a Microbe Gaussian Interaction-Profile-Kernel-based Similarity matrix $Sm_{G}\in R^{N_{m\;}\times N_{m}}$ .

In LCASPMDA, the second type of microbial similarity is measured by the microbial functional similarity in the following way: Firstly, we will construct a microbial protein–protein functional association network and obtain genetic neighbor scores from the STRING database (Szklarczyk et al., 2021). And then, for any two given microbes $m_{i}$ and $m_{j}$ , we will calculate the functional similarity $Sm_{fun}$ ( $m_{i},m_{j}$ ) between them based on the method proposed by Kamneva (2017). Therefore, we can obtain a microbial functional similarity matrix $Sm_{fun}\in R^{N_{m\;}\times N_{m}}$ as well.

Hence, for any two given microbes $m_{i}$ and $m_{j}$ , based on above two kinds of microbial similarities $Sm_{G}\left(m_{i},m_{j}\right)$ and $Sm_{fun}\left(m_{i},m_{j}\right)$ , it is easy to see that we can obtain an integrated microbial similarity $Sm_{sim}\left(m_{i},m_{j}\right)$ according to the following equation (3):


(3) $$Sm_{sim}\left(m_{i},m_{j}\right)=\{\begin{array}{c} \frac{Sm_{G}\left(m_{i},m_{j}\right)+Sm_{fun}\left(m_{i},m_{j}\right)}{2}\;\;if\;Sm_{fun}\neq 0\;\\ Sm_{G}\;\;\left(m_{i},m_{j}\right)\;\;\;otherwise \end{array}$$

#### 2.2.2 Calculation of the integrated similarity of drug

Let *R*(*i*) and *R*(𝑗) denote the *i*th and *j*th rows of the adjacency matrix *A* separately, then in a manner similar to above equations (1), (2), for any two given drugs $d_{i}$ and $d_{j}$ , we can obviously obtain a drug Gaussian Interaction-Profile-Kernel-based Similarity matrix $Sr_{G}\in R^{N_{d\;}\times N_{d}}$ as well.

Besides, in LCASPMDA, the second type of drug similarity is measured by the drug structure similarity proposed by Hattori et al. (2010), and for convenience, for any two given drugs $d_{i}$ and $d_{j}$ , let the drug structure similarity between them be $Sr_{struct}\left(d_{i},d_{j}\right)$ , then it is obvious that we can obtain a drug structure similarity matrix $Sr_{struct}\epsilon \;R^{N_{d\;}\times N_{d}}$ as well.

Hence, for any two given drugs $d_{i}$ and $d_{j}$ , based on above two kinds of drug similarities $Sr_{G}\left(d_{i},d_{j}\right)$ and $Sr_{struct}\left(d_{i},d_{j}\right)$ , it is easy to see that we can obtain an integrated drug similarity $Sr_{sim}\left(d_{i},d_{j}\right)$ according to the following equation (4):


(4) $$Sr_{sim}\left(d_{i},d_{j}\right)=\{\begin{array}{c} \frac{Sr_{G}\left(d_{i},d_{j}\right)+Sr_{struct}\left(d_{i},d_{j}\right)}{2}\;\;if\;Sr_{struct}\neq 0\;\\ Sr_{G}\;\;\left(d_{i},d_{j}\right)\;\;\;otherwise \end{array}$$

#### 2.2.3 Construction of the heterogeneous network

Through combining the adjacency matrix *A* with the integrated microbial similarity matrix $Sm_{sim}$ and the integrated drug similarity matrix $Sr_{sim}$ , it is obvious that we can construct a heterogeneous network of microbes and drugs according to the following equation (5):


(5) $$Y=\left[\begin{array}{cc} Sr_{sim} & A\\ A^{T} & Sm_{sim} \end{array}\right]\in R^{\left(N_{d\;}+N_{m}\right)*\left(N_{d\;}+N_{m}\right)}$$

### 2.3 Feature extraction for nodes in *Y* by LCAT

With the widespread use of GCN, GAT and CAT (Convolutional Attention Networks), researchers have gained some new insight into the limitations of these three kinds of Graph Neural Networks (GNN). For instance, Baranwal et al. (2021) have demonstrated that GCN are significantly data separable when the graph data is neither sparse nor noisy. However, if the graph data is too noisy, the convolution essentially collapses the data to the same value and the GCN may fail. Fountoulakis et al. (2022) have found that GAT exhibits strong differentiability even in noisy datasets. However, under this particular condition, Anderson (2003) pointed out that simple classifiers can also show good results. Therefore, GCN are more beneficial in situations where the noise level is low, and GAT can perform better than GCN in other situations. That is, there is no way to conclude which network structure (GAT or GCN) is the optimal solution in all these two cases. In this research background, Javaloy et al. (2023) have proposed the CAT, and experimentally demonstrated that CAT outperformed GAT with reasonable graph noise, however, it is also not always beneficial to perform convolution before computing attention, which is dependent on the datasets.

It is hard to know before the experiments of cross-validation which of GCN, GAT, or CAT works best. Javaloy et al. (2023) believe that this problem can be solved by learning to interpolate between these three kinds of GNNs, and has proposed a new learnable graph convolutional attention network layer in the following way: for any given node $v_{i}\;$ in Y , let the feature of node $v_{i}\;\;$ be $Y_{i}$ (i.e., the *i*th row of *Y*) and the set of neighboring nodes of $v_{i}\;$ in Y be $N_{v_{i}},$ then based on the following equations (6), (7), the learnable graph convolutional attention network layer can be represented as follows:

(6) $$attention_{v_{i}v_{j}}=\lambda_{1}LeakyRelu\left(a^{T}\left[W\;\;\tilde{Y_{i}}\;\|W\;\;\tilde{Y_{j}}\;\right]\right)\;\;\;\mathrm{where}\tilde{Y_{i}}=\frac{Y_{i}+\lambda_{2}\sum_{j\in N_{v_{i}}\;\;}Y_{j}}{1+\lambda_{2}\left|N_{v_{i}}\right|}\;$$

(7) $$\rho_{v_{i}v_{j}}=\;\;\;softmax\;\left(attention_{v_{i}v_{j}}\right)=\;\;\frac{\exp\left(attention_{v_{i}v_{j}}\right)}{\sum_{k\in N_{v_{i}}}\exp\left(attention_{v_{i}v_{k}}\right)}$$

Here, $attention_{v_{i}v_{j}}$ is the attention score between nodes $v_{i}$ and $v_{j}$ , $\rho_{v_{i}v_{j}}$ is the normalization of $attention_{v_{i}v_{j}}$ , a is the learnable attention vector, and LeakyRelu is the commonly used activation function, $\tilde{Y_{i}}$ and $\tilde{Y_{j}}$ denote the new node features after $Y_{i}$ and $Y_{j}$ have been convolved, $\lambda_{1}$ and $\lambda_{2}$ are trainable values, and W is a trainable weight matrix.

**Figure 2:**
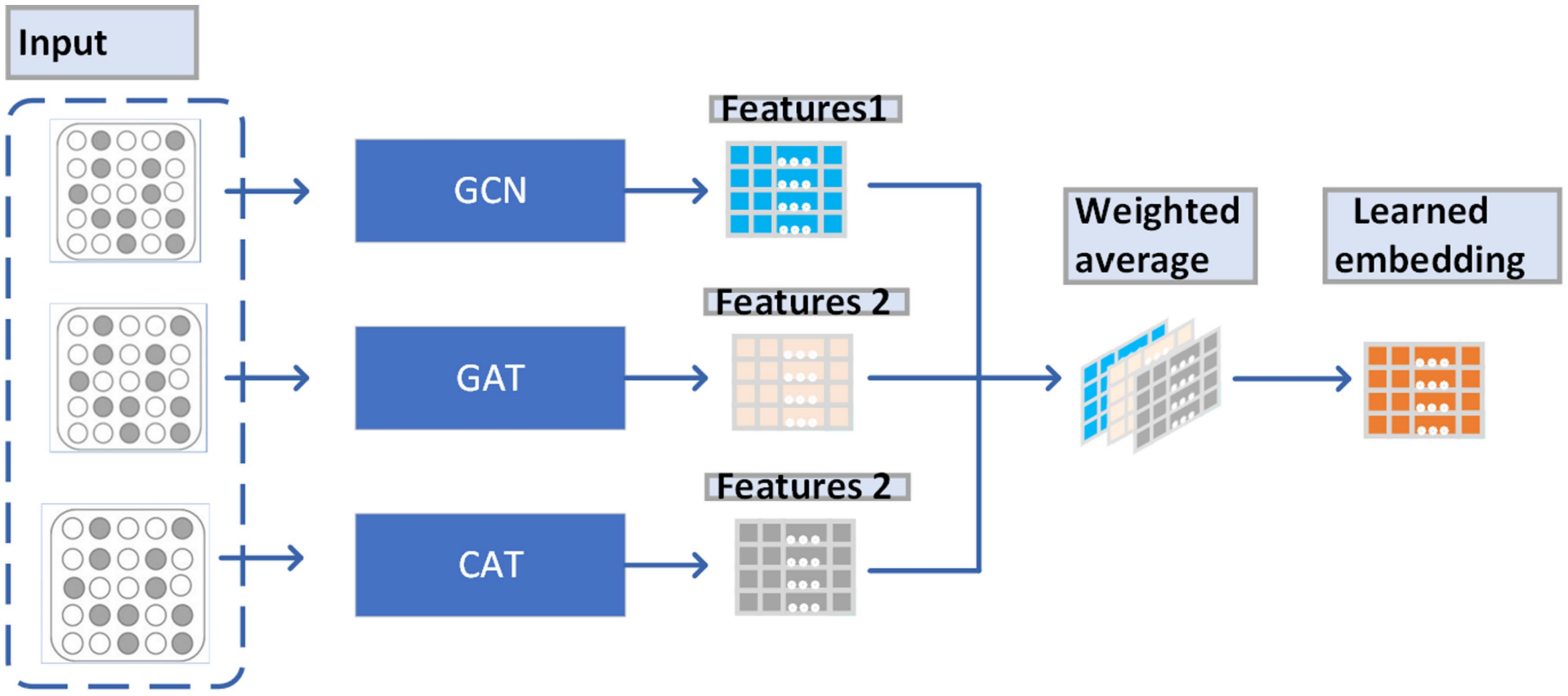
A new perspective on understanding the LCAT principle.

From observing Figure 2, we can understand the way that LACT works as a weighted average of the features obtained by GAT, GCN, and CAT, which enables that the weights of features obtained by GAT, GCN, and CAT can be dynamically adjusted to fit different data sets.

Attention mechanism is an indispensable and complex function of the human brain, as well as an important component of the LCAT. Through the attention mechanism, the human brain can consciously or unconsciously choose from a large number of input information to focus on a small number of useful information. This ensures that people can work in an organized way amid the information bombardment. GAT integrates the attention mechanism with graph neural networks, which has the ability to highlight important information and ignore irrelevant information. The core working principle of GAT is to compute the relationship between nodes by means of the attention mechanism. Among them, we need to clarify the attention vector: in graph neural networks, each node has a vector representing the features of that node. Attention vectors are computed on these feature vectors, which indicates how much attention each node pays to its neighboring nodes. Based on the calculated attention vector, the state of the node can be updated.

Then, after above operations, we can evidently obtain a new feature matrix E ∈ $R^{\left(N_{d\;}+N_{m\;}\right)*F}$ , where each row in *E* represents the newly obtained deep features of nodes in the heterogeneous network Y , and *F* is the dimension of Embedding obtained by the LCAT.

**Figure 3:**
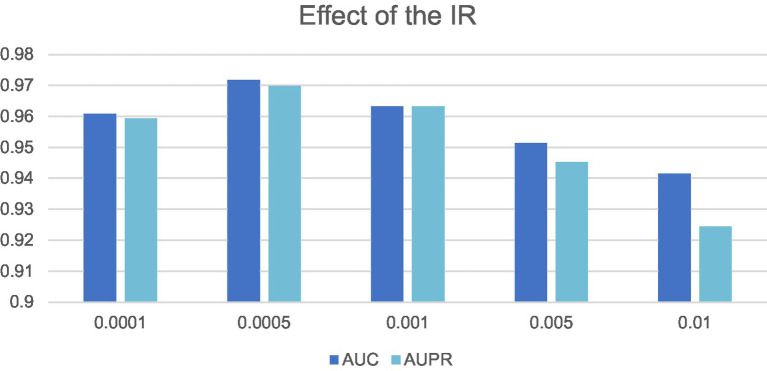
Effects of different *IR* on LCASPMDA.

Through analyses, it is easy to know that the above equation (6) can enable the LCAT to learn to interpolate between the GCN, the GAT and the CAT. For instance, when $\lambda_{1}$ is set to 0, node $v_{i}$ and its neighboring nodes will have the same $\;\rho_{v_{i}v_{j}}$ , and then the LCAT will turn to be a GCN. Additionally, when $\;\lambda_{1}$ =1 and $\lambda_{2}$ =0, then the LCAT will be a GAT. Moreover, when $\lambda_{1}$ and $\lambda_{2}$ are both set to 1, the LCAT will be a CAT. In this manuscript, as shown in Figure 3, we have also proved that LCAT is able to integrate the advantages of all these three kinds of GCNs and can achieve the best performance in different datasets.

### 2.4 Selection of negative samples for training the MLP classifier

#### 2.4.1 The MLP classifier

MLP is a powerful tool for classification tasks, and its superiority has been proven in common binary classification tasks. In LCASPMDA, we will adopt the MLP classifier as the final decoder in the following way: firstly, the embedding of microorganisms and drugs obtained by LCAT will be taken as inputs of the MLP classifier. And then, the MLP classifier will implement the element-wise multiplication operation on these embeddings. Finally, the predicted score matrix of potential associations between microorganisms and drugs will be obtained after the processing of the activation function. For any given microorganism $m_{i}$ and drug $d_{j}$ , the final predicted score of potential association between them will be calculated according to the following equation (8):


(8) $$Score_{ij}=\;\;Sigmoid\left(W_{2}\left(Rule\left(W_{1}\left(E_{m_{i}}⊙E_{d_{j}}\right)\right)\right)\right)$$

Where $Score\in R^{N_{d}\times N_{m}}$ is the final predicted score matrix. $\;E_{m_{i}}$ and $\;E_{d_{j}}$ are embedding of $m_{i}$ and $d_{j}$ obtained by LCAT respectively, $W_{1}$ and $W_{2}$ are trainable matrices, and the ⨀ operation is the element-wise multiplication. Let *F* be the dimension of Embedding obtained by the LCAT, then there is $E_{m_{i}}\in R^{F}$ , $E_{d_{j}}\in R^{F}$ , $W_{1}\in R^{F\times F}$ and $W_{2}\in R^{1\times F}$ . In addition, *Rule* and *Sigmoid* are activation functions adopted in the MLP classifier.

#### 2.4.2 Self-paced iterative sampling ensemble

Studies have demonstrated that the model performance decreases on datasets with imbalanced positive and negative samples (Liu et al., 2020). The imbalance of positive and negative samples poses a considerable challenge to the training of classifiers. In simple terms, the unbalance of samples can cause serious deviations in the classification model, but it cannot be seen from some common metrics, for example, in the case where the number of positive samples is much larger than the number of negative samples, the trained model may already have a very high accuracy, but we can classify all the negative samples as false positive in such a case to also have a very high accuracy. In two well-known microbe-drug association databases such as the MDAD and the aBiofilm, all microbe-drug pairs with known associations are viewed as positive samples, while the remaining microbe-drug pairs are regarded as candidate negative samples. In all these two well-known databases, the number of candidate negative samples far exceeds the number of positive samples. In previous studies, many researchers have found this point, so they always choose the under-sampling method to balance the samples in order to ensure the balance of the dataset, and the commonly used method is the random under-sampling method. In this method, researchers will randomly draw the same number of negative samples as positive samples in the candidate negative sample set, thus ensuring that the ratio of positive to negative samples is 1:1. In this method, since the negative samples are selected randomly, the specificity of the negative samples is not fully considered, it may result in the loss of informative negative samples and introduction of meaningless samples at the same time. Selecting informative samples from the candidate negative samples is a challenging task, and in LCASPMDA, we will introduce the Self-Paced Iterative Sampling Ensemble strategy to pick out the informative negative samples (Liu et al., 2020).

The Self-Paced Iterative Sampling Ensemble strategy proposed the concept of hardness function *H*, according to which the candidate negative samples were categorized into three categories such as the trivial samples, the noise samples and the edge samples, respectively. Among them, the noise samples have larger values of *H*, which means it may be a false negative sample. The trivial samples have smaller *H* values, which means it can be well classified by the prediction model. In LCASPMDA, we only need to keep a small portion of the trivial samples because they have been well learned. The remaining edge samples are the most informative samples in our training process, which symbolize the decision boundary of the prediction model. Obviously, expanding the proportion of edge samples on the dataset will benefit to improve the performance of the prediction model. In LCASPMDA, we will adopt the Self-Paced Iterative Sampling Ensemble strategy to pick out the informative negative samples according to the following steps:

Step 1: In LCASPMDA, the predicted values of all associations between microbes and drugs will be obtained by using the MLP classifier.

Step 2: the hardness function in the Self-Paced Iterative Sampling Ensemble strategy is defined by the following equation (9):


(9) $$H\left(x,\;y,\;F\right)=\;\;{\left(F\left(x\right)-y\right)}^{2}$$

Here, F(x) represents the predicted score value obtained by the MLP classifier for the sample *x*, and *y* is the original label value of the sample *x*.

Step 3: All candidate negative samples are classified into *k* buckets based on the hardness function according to the following equation (10):


(10) $$B_{l}=\left\{\left(\mathrm{x,}\;y\right)|\frac{l-1}{k}\leq H\left(x,\;y,\;F\right)\leq \frac{l}{k}\;\right\},H\in \left[0,\;1\right],$$

Here, *k* is the number of buckets and is a hyperparameter. $B_{l}$ represents the negative sample of the lth bucket.

Step 4: Adopting the Self-Paced Iterative Sampling Ensemble strategy to select different numbers of negative samples from *k* buckets to form the negative sample set for the next iteration of training. Let $N_{B_{l}}$ is the number of negative samples selected in the $B_{l}$ bucket, then $N_{B_{l}}$ can be calculated according to the following equation (11):


(11) $$\{\begin{array}{c} h_{l\;\;}=\;\;\;\underset{x_{ij}\in B_{l}}{\sum }H\left(x_{ij},y_{ij},F\right)\cdot {\left|B_{l}\right|}^{-1}\\ \beta \;\;\;=\;\;-\log\frac{n-i}{n}\\ W_{l}=\;\;{\left(h_{l\;\;}+\beta \;\right)}^{-1}\\ N_{B_{l}}=\frac{W_{l}}{\sum_{t}W_{t}}\;\cdot \left|P\right|\;t=1,\;.....,\;k \end{array}$$

Here, $h_{l\;\;}$ represents the average hardness value of the *l*th bucket, *n* is the number of epochs for which the model is ready to be trained and i is the current number of iterations, β is the self-paced factor, $W_{l}$ denotes the normalized sampling weight of the *l*th bucket, |P| is the number of positive samples, and l=1,··⋯,k .

Step 5: Randomly selecting $N_{B_{l}}\;$ negative samples from the *l*th bucket, and gathering all the selected negative samples to form a new negative sample set. The set of negative samples selected by the Self-Paced Iterative Sampling Ensemble and all the positive samples are combined into a new training set to train the MLP classifier and proceed to the next iteration.

While implementing above strategy, we will update the hardness value at each iteration in order to generate the most informative samples. The self-paced factor *β* is the focus of the above strategy. The role of the self-paced factor *β* has been demonstrated experimentally in SPE (Liu et al., 2020). Considering that as the training iterates, the number of trivial samples grows, then we need to reduce the weight value of the bucket with a large number of samples so that we can focus more on samples with higher hardness values. Therefore, in LCASPMDA, we will introduce a self-paced factor *β* growing from zero to infinity, and the growth of the self-paced factor *β* will be controlled by using a logarithmic function at the same time.

#### 2.4.3 Loss function

Since the association prediction problem belongs to the binary classification problem, in which the binary cross entropy has shown excellent performance, then in LCASPMDA, we will as well adopt the binary cross entropy function as the loss function of the MLP classifier, which is defined as follows:


(12) $$L=-\underset{\left(i,j\right)\in z^{+}\cup z^{-}\;}{\sum }\;\;z_{ij}\log Score_{ij}+\left(1-z_{ij}\right)\log\left(1-Score_{ij}\right)\;$$

In LCASPMDA, we will consider each microbe-drug pair (*i*, *j*) as an independent microbe-drug sample. Besides, in above equation (12), $z^{+}$ denotes a subset of positive samples in the training sample and $z^{-}$ represents a subset of negative samples, and for any given microbe-drug pair (*i*, *j*) belonging to $z^{+}$ , we will set its base truth value $z_{ij}$ to 1, while for any given microbe-drug pair (*i*, *j*) belonging to $z^{-}$ , we will set its base truth value $z_{ij}\;$ to 0. Moreover, *Score_ij_* represents the predicted score of the association between the *i*th microbe and the *j*th drug in the final score matrix obtained by the MLP classifier.

Finally, we will put the new features extracted by LCAT into the MLP classifier trained by the self-paced iterative sampling ensemble strategy to obtain the final output of our prediction model. Obviously, the MLP classifier will generate the predicted score of potential association between each pair of microbe and drug, which can help us discover the criticality of hidden microbe-drug associations.

## 3 Experiments and results

In this section, we verified the prediction performance of LCASPMDA based on the framework of 5-fold cross-validation. In experiment, for any given newly-downloaded microbe-drug dataset, we will divide all microbe-drug pairs equally into five parts, and used one part at each time as the test set and the rest as the training set. Moreover, we will introduce the AUPR and the AUC values as the evaluation metrics to measure the performance of the model. In this section, to demonstrate the superiority of the LCASPMDA model, we will Comparison with baseline methods. Additionally, in order to improve the performance of LCASPMDA, we will first study the role of various parameters inside the model, and then, we will do ablation experiments to examine the contribution of the Self-Paced Iterative Sampling Ensemble strategy and the LCAT to the model. Finally, to prove the validity of our model, we will select some drugs from inside the database of MDAD to do case studies.

### 3.1 Comparison with baseline methods

In order to verify the prediction performance of LCASPMDA, in this section, we will compare it with the following five representative competing methods based on the databases of MDAD and aBiofilm respectively:

- GCNMDA (Long et al., 2020a): in which, a graph convolutional network framework integrated with conditional random fields was proposed to infer potential associations between microbes and drugs.
- GSAMDA (Tan et al., 2022): which utilized graph attention networks and sparse auto-encoders to capture topological features and attribute features of nodes in a newly-constructed microbe-drug heterogeneous network first, and then, computed the likelihood of potential associations between microbe-drug pairs by leveraging these newly-captured features of microbes and drugs.
- MDASAEA (Fan et al., 2023): which predicted latent microbe-drug associations by combining the self-sparse encoders and the multi-head attention networks.
- LRLSHMDA (Wang et al., 2017): which employed the Laplace-regularized least squares classifier, a semi-supervised computational model, for predicting possible microbe-disease associations.
- NTSHMDA (Luo and Long, 2020): in which, an improved randomized wandering algorithm was used to infer potential microbe-disease associations by integrating topological similarities of nodes in a newly-constructed microbe-drug heterogeneous network.

**Figure 4:**
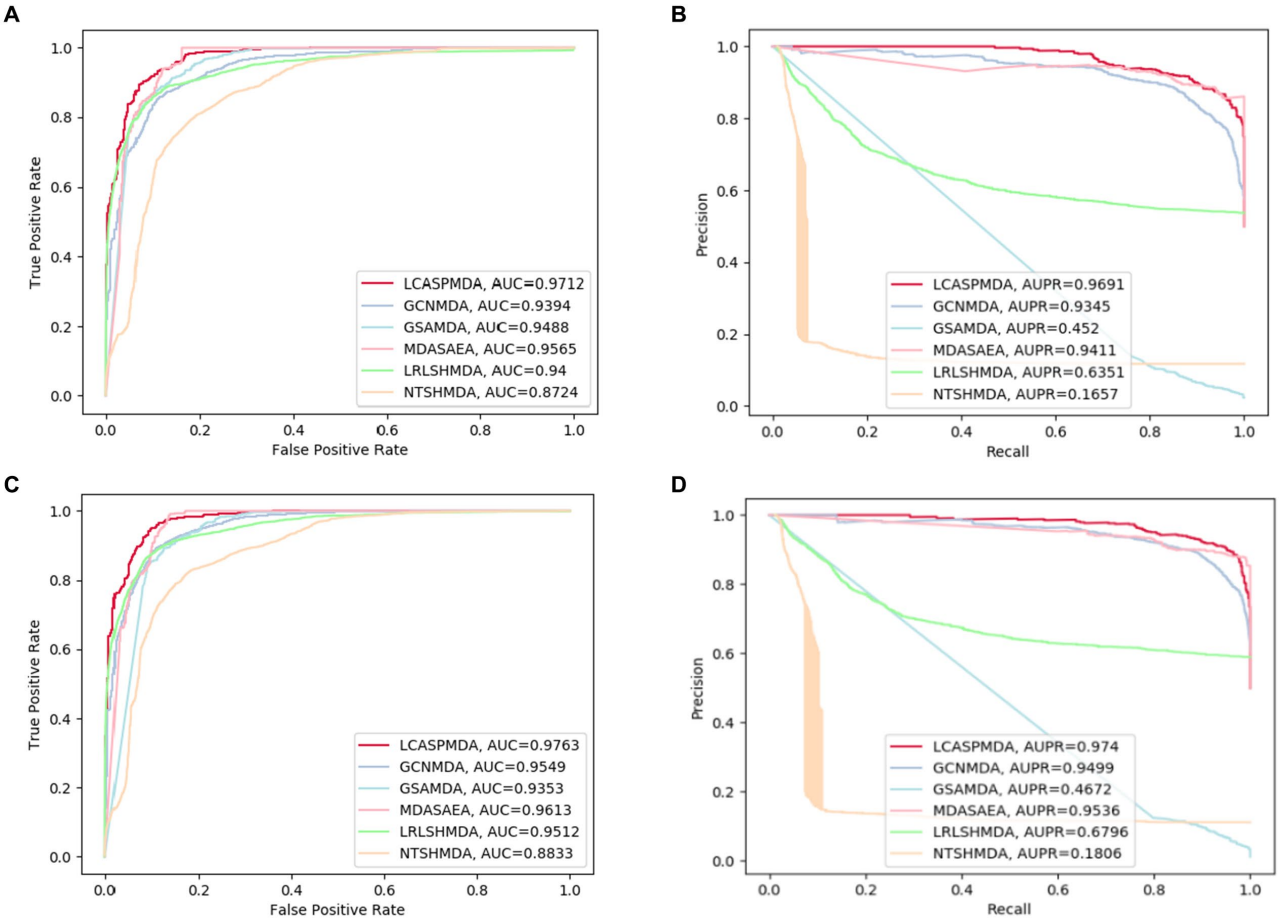
ROC and PR curves achieved by LCASPMDA and state-of-the-art methods based on MDAD and aBiofilm separately. **(A)** ROC curves based on MDAD, **(B)** PR curves based on MDAD, **(C)** ROC curves based on aBiofilm, **(D)** PR curves based on aBiofilm.

**Figure 5:**
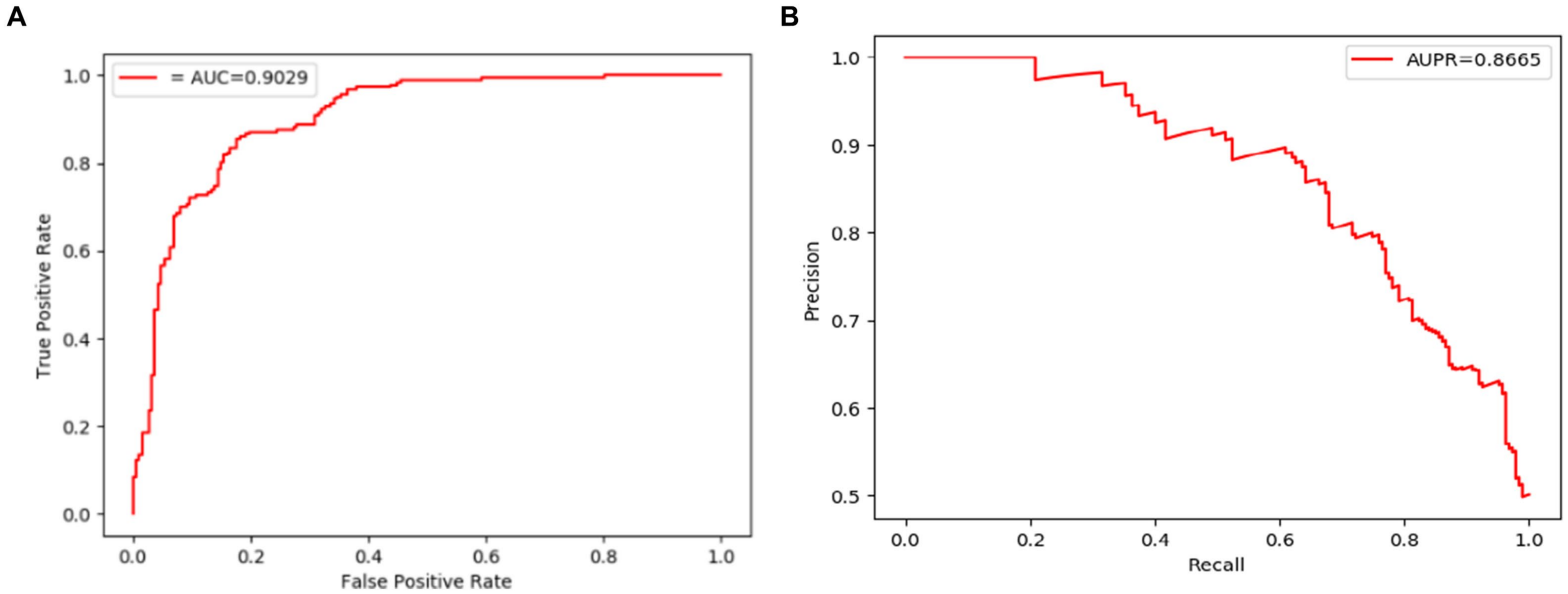
ROC and PR curves achieved by LCASPMDA based on the DrugVirus database. **(A)** ROC curves based on DrugVirus, **(B)** PR curves based on DrugVirus.

The comparison results are shown in Tables 2, 3. And in addition, we illustrate the optimal ROC curves and PR curves of these six competing methods, based on the databases of MDAD and aBiofilm respectively, in Figure 4 to highlight the superiority of LCASPMDA. Finally, in order to better show the prediction performance of LCASPMDA, we further conducted intensive comparison experiments based on multiple metrics, in addition to the commonly used metrics such as the AUC and the AUPR, under the MDAD database and the aBiofilm database, respectively. And the comparison results were shown in Table 4. Besides, we provided the experimental results of LCASPMDA based on the DrugVirus database in Figure 5 as well.

**Table 2:** Performance comparison between baseline methods and LCASPMDA on MDAD under the framework of 5-fold cross-validation.

Methods	AUC	AUPR
GCNMDA	0.9390 ∓ 0.0014	0.9346 ∓ 0.0105
GSAMDA	0.9456 ∓ 0.0007	0.4513 ∓ 0.0008
MDASAEA	0.9547 ∓ 0.0015	0.9406 ∓ 0.0032
LRLSHMDA	0.9397 ∓ 0.0031	0.6347 ∓ 0.0016
NTSHMDA	0.8683 ∓ 0.0021	0.1542 ∓ 0.0115
**LCASPMDA**	**0.9703** ∓ **0.0109**	**0.9674** ∓ **0.0117**

The bold values are predicted scores achieved by LCASPMDA.

**Table 3:** Performance comparison between baseline methods and LCASPMDA on aBiofilm under the framework of 5-fold cross-validation.

Methods	AUC	AUPR
GCNMDA	0.9537 ∓ 0.0022	0.9376 ∓ 0.0123
GSAMDA	0.9251 ∓ 0.0101	0.4649 ∓ 0.0022
MDASAEA	0.9604 ∓ 0.0007	0.9534 ∓ 0.0032
LRLSHMDA	0.9510 ∓ 0.0007	0.6747 ∓ 0.0049
NTSHMDA	0.8811 ∓ 0.0022	0.1602 ∓ 0.0204
**LCASPMDA**	**0.9742** ∓ **0.0121**	**0.9720** ∓ **0.0120**

The bold values are predicted scores achieved by LCASPMDA.

**Table 4:** Performance comparison between baseline methods and LCASPMDA base on the MDAD and the aBiofilm databases.

Datadabse	Performance Metrics	LCASPMDA	GCNMDA	GSAMDA	MDASAEA	NTSHMDA	LRLSHMDA
MDAD	ACC	0.9146 ∓0.0231	0.7914 ∓ 0.0149	0.5766 ∓ 0.0156	0.9045 ∓0.0107	0.9657 ∓0.0317	0.9700 ∓0.0227
	F1-SCORE	0.9144 ∓0.0159	0.7500 ∓ 0.0218	0.7707 ∓ 0.0233	0.9270 ∓ 0.0158	0.3205 ∓ 0.0247	0.3009 ∓ 0.0177
	MCC	0.8426 ∓ 0.0478	0.6101∓ 0.0359	0.2881 ∓ 0.0558	0.8415 ∓ 0.0287	0.3918 ∓ 0.0256	0.3344 ∓ 0.0258
aBiofilm	ACC	0.8932 ∓0.0257	0.8198 ∓0.0186	0.5214 ∓ 0.0378	0.8898 ∓0.0145	0.9592 ∓ 0.0309	0.9693 ∓ 0.0108
	F1-SCORE	0.8954 ∓ 0.0186	0.7634 ∓ 0.0274	0.7554 ∓ 0.0246	0.8845 ∓0.0203	0.2262 ∓ 0.0256	0.2367 ∓0.0214
	MCC	0.7964 ∓0.0254	0.6275 ∓ 0.0478	0.2382 ∓0.0742	0.7335 ∓ 0.0288	0.3329 ∓0.0312	0.3427 ∓ 0.0212

From above two tables, it can be seen that LCASPMDA can achieve the best prediction performance among these six competing methods. And the AUC and AUPR values of LCASPMDA on MDAD are 0.9703 ∓ 0.0109 and 0.9674 ∓ 0.0117, respectively.

From observing Figure 4, we can clearly see the superiority of LCASPMDA. Among these six competing methods, MDASAEA is only method that can achieve better performance than LCASPMDA in terms of ROC curve, but in terms of PR curve, the PR values of the GSAMDA on MDAD and aBiofilm are only 0.4513 ∓ 0.0008 and 0.4649 ∓ 0.0022, respectively, which are quite lower than that of LCASPMDA. Through analysis, the reason that why GSAMDA can only achieve such a lower PR value is that the extreme imbalance in the proportion of positive and negative samples causes the model to be biased toward making predictions with the majority class, i.e., the negative examples, resulting in lower precision and recall for positive examples. However, LCASPMDA selects information-rich negative samples by the Self-Paced Iterative Sampling Ensemble method while balancing the ratio of positive and negative samples, which ensures that satisfactory PR values can be obtained. Besides, the PR curve achieved by NTSHMDA is the strangest one, first of all, it seems to be relatively coarse, after analysis, we find the reason is that the model floats a lot in a time period and the time gap is very short. Secondly, the AUPR values achieved by NTSHMDA are only 0.1542 ∓ 0.0115 and 0.1602 ∓ 0.0204 on MDAD and aBiofilm separately, after analysis, we find the reason is that it does not have a regularization operation to prevent the model from over-fitting, and at the same time, it does not use a deep learning algorithm with a loss function to point out a correct direction of learning. However, LCASPMDA uses the dropout method to prevent the model from over-fitting during model training, and at the same time, chooses the cross-entropy function, which is most suitable for binary classification tasks, as the loss function. Meanwhile, we also find that NTSHMDA achieved the lowest AUC and AUPR values compared to other models using deep learning algorithms. From observing Table 4, it is easy to see that LCASPMDA significantly outperformed all these baseline models as a whole. Through analysis, we found that the main reason is due to the extreme imbalance in the proportion of positive and negative samples of other models (Except for GCNMDA and MDASAEA) as well as the excellent model architecture adopted by LCASPMDA. Moreover, it is well known that the metrics of AUC and ACC are insensitive to the proportions of positive and negative samples, however the rest of the metrics are very sensitive to their proportions, and in addition, MCC is a more comprehensive performance metric that only scores high when good results are obtained for these four metrics (True Positive, True Negative, False Positive, False Negative), that is the reason why NTSHMDA and LRLSHMDA performed well in the ACC metric but poorly in the rest of the metrics. Besides, LCASPMDA achieved better performance than GCNMDA, the reason is that although these two models can maintain the balance of positive and negative sample ratios, but LCASPMDA utilizes an innovative model LCAT to extract node features, which is more effective than the GCN adopted by GCNMDA.

### 3.2 Parameter analysis

**Figure 6:**
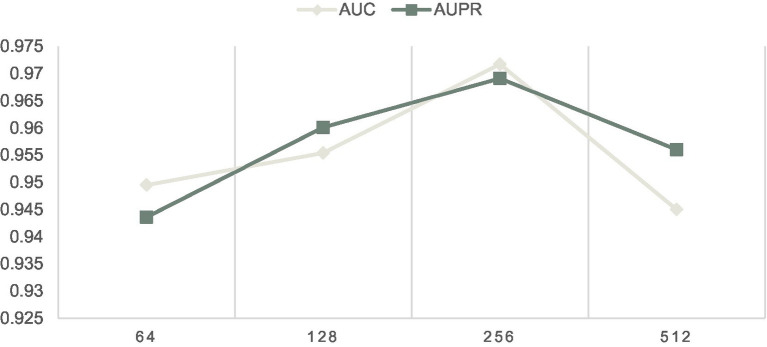
Effects of different *Out-dimension* on LCASPMDA.

In this section, we will evaluate the effect of two important parameters, including the parameters *IR* and *Out-dimension* that denote the learning rate of our model and the number of embedding dimension of the LCAT separately, on LCASPMDA, based on the MDAD database. Through observing Figure 3, we can clearly know that LCASPMDA performs best when the learning rate *IR* is set to 0.0005. When we explore the impact of the parameter *Out-dimension*, we set the *Out-dimension* to {64, 128, 256, 512} respectively, and show the experimental results in Figure 6. It is obvious that the AUC value of LCASPMDA peaks when *Out-dimension* is set to 256, and the AUC value tends to decrease when it exceeds 256. Based on above analysis, we will finally set the parameter *IR* to 0.0005 and the parameter *Out-dimension* to 256 in experiments.

### 3.3 Ablation study

**Figure 7:**
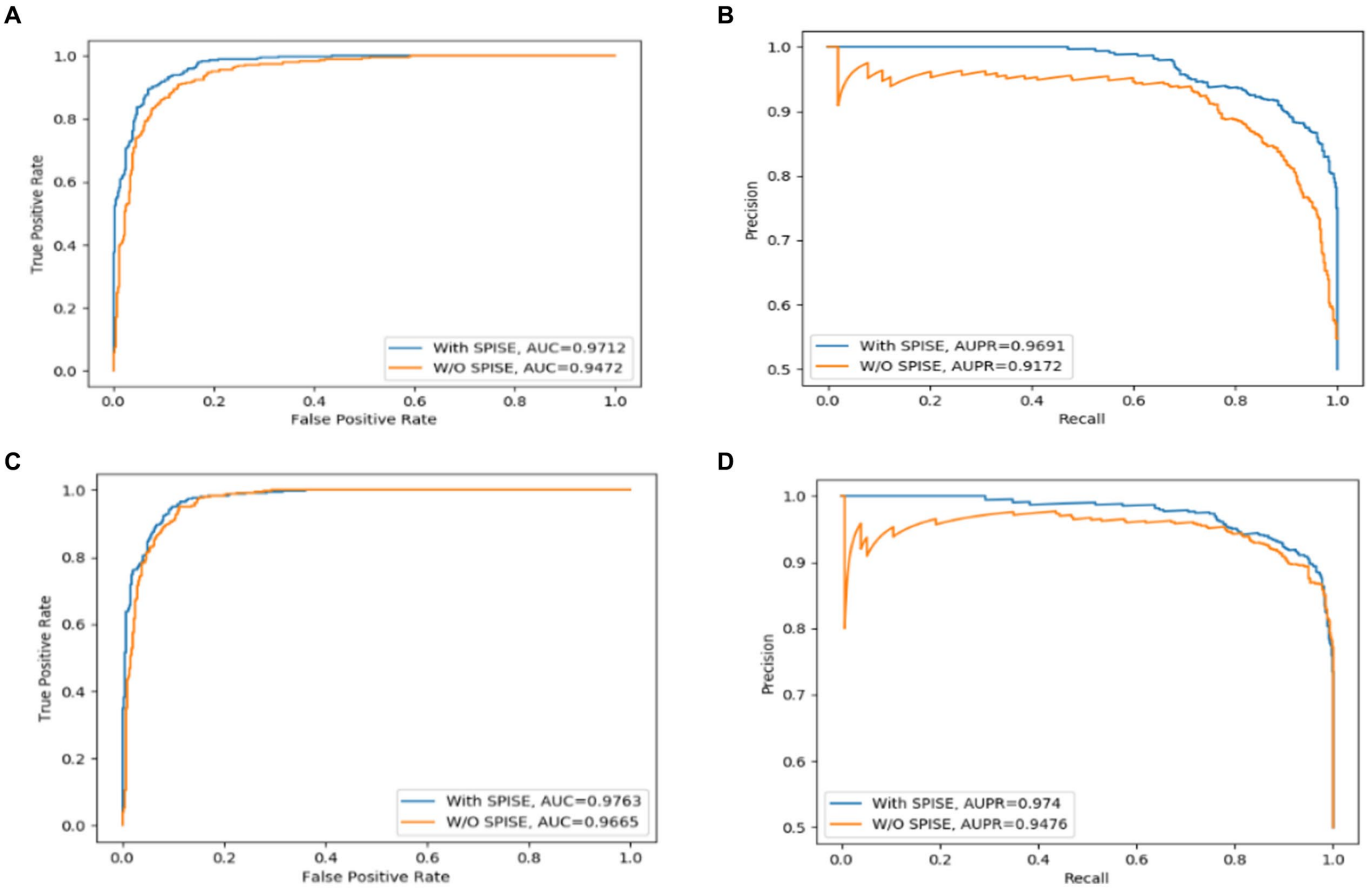
SPISE can improve the prediction performance of LCASPMDA. **(A)** ROC curves based on MDAD, **(B)** PR curves based on MDAD, **(C)** ROC curves based on aBiofilm, **(D)** PR curves based on aBiofilm.

**Figure 8:**
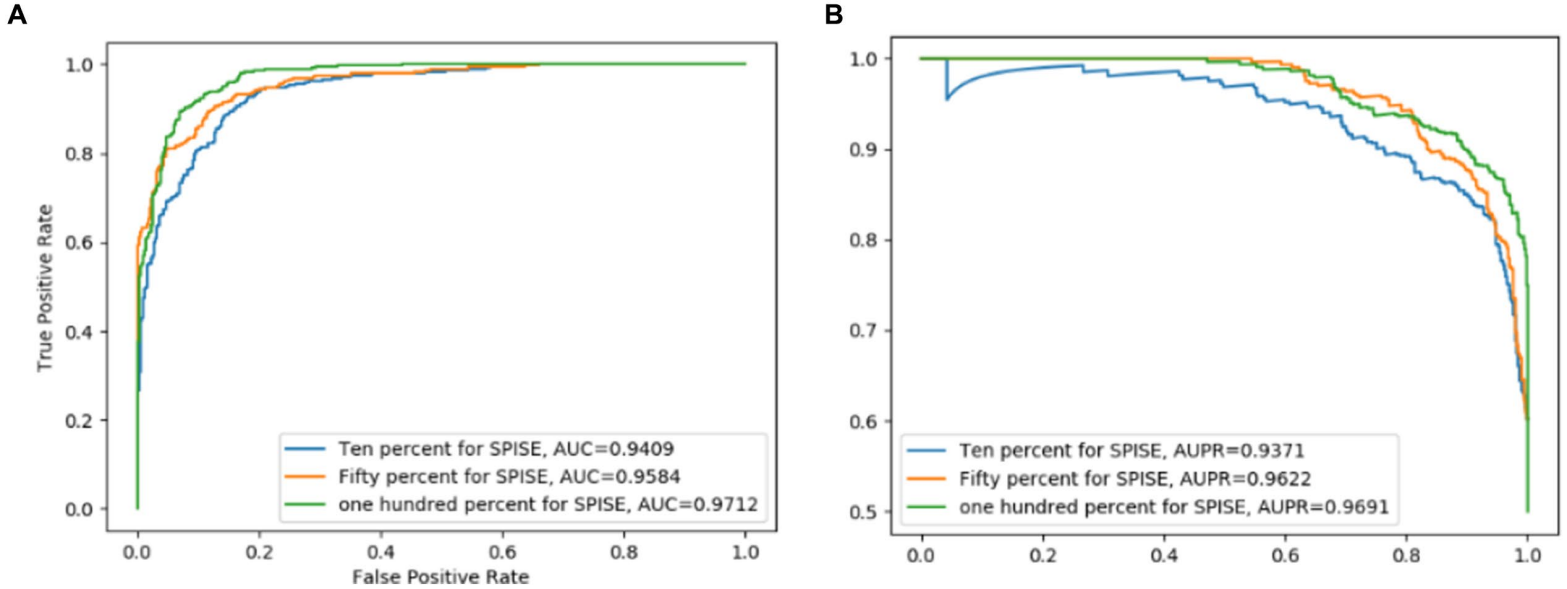
SPIE has an important effect on the overall model performance of LCASPMDA. **(A)** ROC curves, **(B)** PR curves.

**Figure 9:**
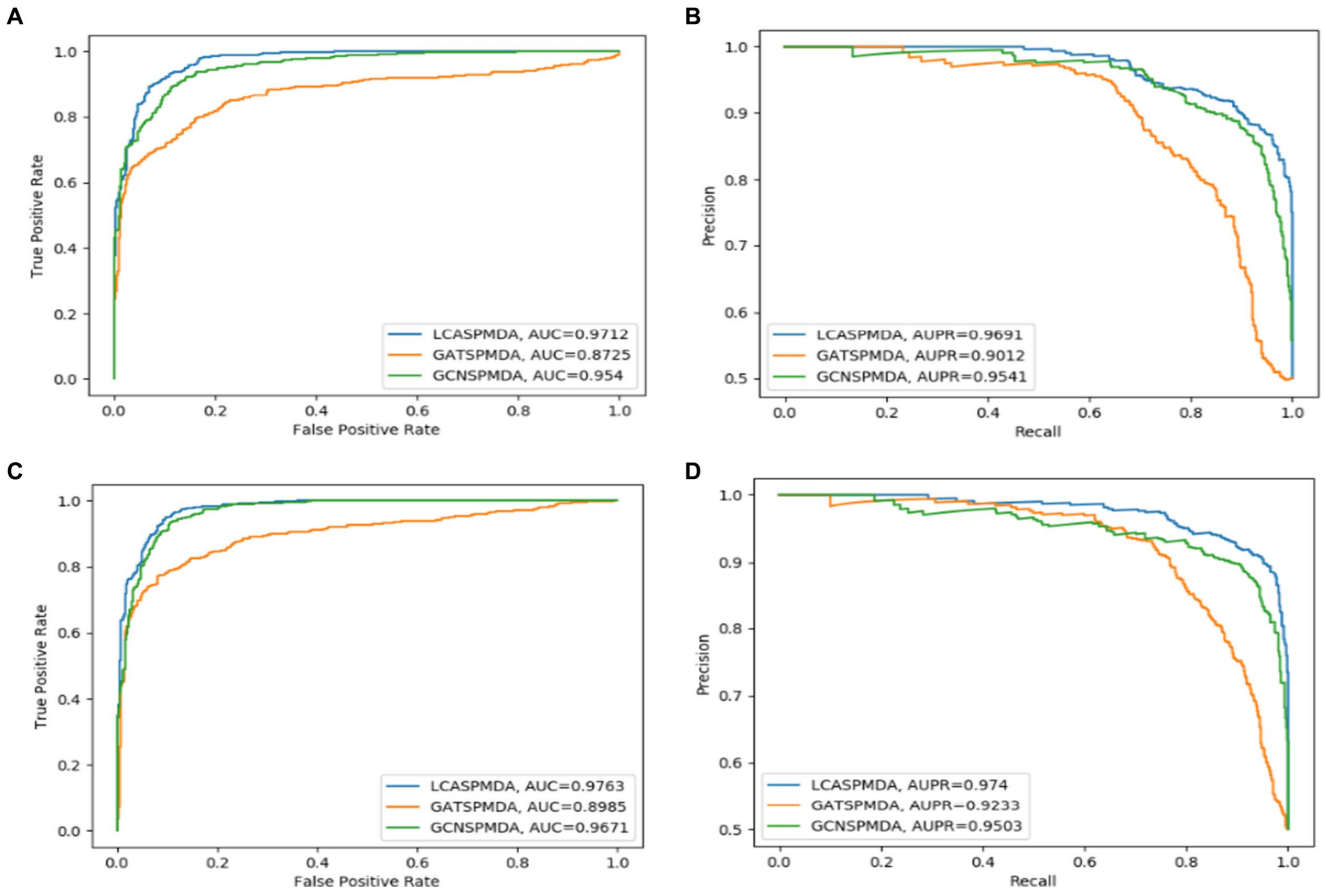
LCAT can improve the prediction performance of LCASPMDA. **(A)** ROC curves based on MDAD, **(B)** PR curves based on MDAD, **(C)** ROC curves based on aBiofilm, **(D)** PR curves based on aBiofilm.

The Self-Paced Iterative Sampling Ensemble strategy (hereinafter referred to as SPISE) is the core part of LCASPMDA, which focuses on how to obtain a balanced dataset in an unbalanced set of positive and negative samples through a special negative sampling method while ensuring that the negative samples have a large amount of information. In order to evaluate the impact of SPISE on the performance of LCASPMDA, we first conducted an ablation study in this section. And then, considering that SPISE is a pivotal component of the LCASPMDA framework, to fully ascertain the effectiveness of SPISE, we further conducted an additional evaluation by varying the proportions of negative samples selected by SPISE, and in the experiment, part of the negative samples were selected by SPIE, and the rest were selected by random. In addition, we also conducted experiments by replacing the LCAT in LCASPMDA with the GAT and the GCN, respectively. As shown in Figures 7–9, it is easy to see that adopting the SPISE can improve the prediction performance of LCASPMDA observably, and simultaneously, adopting the LCAT can achieve better performance than adopting the GAT and the GCN in LCASPMDA as well.

### 3.4 Case study

To further validate the ability of LCASPMDA in predicting unknown associations between microorganisms and drugs, we conducted case studies, respectively, based on two drugs, including the Ciprofloxacin and the Pefloxacin, which are commonly used in MDAD. We trained the models based on the MDAD database. Specifically, for each selected target drug, all known microbe-drug associations were set to be unknown, and then all candidate microbes were ranked in the descending order based on predicted scores obtained by LCASPMDA. In experiment, for any given drug, we would choose the top 20 related microorganisms predicted by LCASPMDA, and verified that whether these predicted microbes had already been reported to be associated with the given drug in the PubMed literatures.

As for the Ciprofloxacin, it is a fluoroquinolone-containing drug with a high potential for antibacterial activity, and commonly used in the treatment of joint infections, respiratory infections and other treatments. Ciprofloxacin has broad-spectrum antimicrobial activity, with strong bactericidal effect against *Pseudomonas aeruginosa*, *Staphylococcus aureus* and other common pathogenic bacteria. Numerous experiments have also confirmed the close relationship between Ciprofloxacin and human microorganisms. For example, Hacioglu et al. (2019) discovered and validated Ciprofloxacin as an active drug against *Candida albicans*. Besides, Trinh et al. (2017) demonstrated that the combination of ceftriaxone and Ciprofloxacin was the most effective treatment for foodborne Vibrio traumaticus. Moreover, Cho et al. (2019) found that *Mycobacterium avium* was highly sensitive to Ciprofloxacin. Table 5 shows the predicted results of the top 20 microorganisms associated with Ciprofloxacin, 17 of which have been demonstrated in the PubMed literatures.

**Table 5:** The top 20 predict Ciprofloxacin-associated microbes.

Microbe	Rank	Evidence	Microbe	Rank	Evidence
*Escherichia coli*	1	PMID: 26607324	*Stenotrophomonas maltophilia*	11	PMID: 28488744
*Pseudomonas aeruginosa*	2	PMID: 30605076	*Bacillus subtilis*	12	PMID: 15194135
*Staphylococcus aureus*	3	PMID: 32488138	*Candida* spp.	13	Unconfirmed
*Burkholderia cepacia*	4	PMID: 10091030	*Aeromonas hydrophila*	14	PMID: 24242249
*Klebsiella planticola*	5	PMID: 25465871	*Burkholderia multivorans*	15	PMID:19633000
*Burkholderia cenocepacia*	6	PMID:27799222	*Streptococcus pneumoniae*	16	PMID: 15155208
*Klebsiella pneumoniae*	7	PMID:27257956	*Micrococcus luteus*	17	PMID:16340189
*Listeria monocytogenes*	8	PMID:28355096	Enteric bacteria and other eubacteria	18	PMID: 27436461
*Vibrio harveyi*	9	PMID:27247095	*Streptococcus epidermidis*	19	Unconfirmed
*Acinetobacter baumannii*	10	PMID:25147676	*Kocuria rhizophila*	20	Unconfirmed

Pefloxacin is a broad-spectrum quinolone antibiotic with significant bactericidal effects against a wide range of Gram-negative and Gram-positive bacteria. For instance, Wang et al. (2021) studied the resistance of *Escherichia coli* strains to Pefloxacin and found that the resistance rate was as high as 68.8%, which provided new clues for the study of genetic and epidemiological characterization of urinary tract infections after renal transplantation. Moin et al. (2020) detected the susceptibility of *Salmonella enterica* thermophila to fluoroquinolones with the Pefloxacin disk diffusion test. Abdoulaye et al. (2018) isolated 126 bacterial strains from patients with surgical site infections (ISO) at the National Hospital of Niamey and found them to be resistant to different fluoroquinolones (e.g., Pefloxacin and nalidixic acid) to varying degrees. Table 6 illustrates the predicted results for the top 20 Pefloxacin-associated microorganisms, 14 of which have been proved in the PubMed literatures.

**Table 6:** The top 20 predict Pefloxacin-associated microbes.

Microbe	Rank	Evidence	Microbe	Rank	Evidence
*Staphylococcus aureus*	1	PMID: 2258345	Human Immunodeficiency Virus 1	11	PMID:9495677
*Staphylococcus epidermis*	2	PMID: 2640275	*Clostridium perfringens*	12	Unconfirmed
*Vibrio harveyi*	3	Unconfirmed	Enteric bacteria and other eubacteria	13	Unconfirmed
*Bacillus subtilis*	4	PMID: 12024980	*Pseudomonas aeruginosa*	14	PMID: 1645509
*Staphylococcus epidermidis*	5	PMID: 26607324	*Burkholderia cenocepacia*	15	Unconfirmed
*Burkholderia thailandensis*	6	Unconfirmed	*Mycobacterium smegmatis*	16	PMID: 25379514
*Listeria monocytogenes*	7	PMID: 2504545	Human immunodeficiency virus	17	PMID: 9495677
*Enterococcus faecalis*	8	PMID: 2258345	*Micrococcus luteus*	18	PMID:16340189
*Burkholderia pseudomallei*	9	PMID: 19121669	*Actinoplanes missouriensis*	19	Unconfirmed
*Streptococcus pneumoniae*	10	PMID: 20384283	*Salmonella enterica*	20	PMID: 28948961

## 4 Conclusion

We are committed to discovering more potential microbe-drug associations and making our contribution to the protection of human health. In this paper we have proposed a novel prediction model called LCASPMDA by combining a learnable graph convolution attention network, a Self-Paced Iterative Sampling Ensemble strategy and a multi-layer perceptron. Comparative experiments and case studies show that LCASPMDA can achieve excellent prediction performance. And at the same time, there are still areas where it can be improved, for example, LCASPMDA does not collect or use any actual negative samples. Secondly, using MLP to generate new microbe-drug association matrices may provide useless association information. Thirdly, the parameters used in MLP and LCAT may not be optimal and may even be biased, and the lack of negative samples may significantly affect the predictive performance of LCASPMDA. Therefore, on the one hand, it is crucial to obtain negative samples from biomedical databases and literature. On the other hand, developing computational methods to generate high-quality negative samples is another option to address this issue. In addition, it is noted that selected negative samples can achieve significant performance improvements in the area of protein-RNA interaction identification as well. Meanwhile, we can introduce some new mechanisms such as the attention mechanism, spatial convolution mechanism and so on to improve the performance of the model.

## Data availability statement

The original contributions presented in the study are included in the article/Supplementary material, further inquiries can be directed to the corresponding authors.

## Author contributions

ZY: Data curation, Methodology, Resources, Software, Writing – original draft, Writing – review & editing. LW: Conceptualization, Funding acquisition, Investigation, Methodology, Project administration, Supervision, Writing – original draft. XZ: Data curation, Formal analysis, Validation, Writing – original draft. BZ: Methodology, Supervision, Validation, Writing – review & editing. ZZ: Investigation, Methodology, Supervision, Visualization, Writing – review & editing. XL: Methodology, Software, Supervision, Validation, Writing – review & editing.

## Footnotes

^1^ https://github.com/longyahui/GCNMDA
